# Understanding antibiotic knowledge, attitudes, and practices: a cross-sectional study in physicians from a Colombian region, 2023

**DOI:** 10.1186/s12909-024-05354-w

**Published:** 2024-04-08

**Authors:** Juan Camilo Morales Taborda, Juan Carlos Montaño Guzmán, Luis Felipe Higuita-Gutiérrez

**Affiliations:** 1 Facultad de medicina, Universidad Cooperativa de Colombia, Medellín, Colombia; 2 Escuela de microbiología, Universidad de Antioquia, Medellín, Colombia

**Keywords:** Health knowledge, Attitudes, Practice, Survey, Antibiotics, Physicians

## Abstract

**Background:**

Antibiotic resistance has been identified as a global health threat. Knowledge, attitudes, and inappropriate prescription practices of antibiotics by physicians play a crucial role in this problem. In Colombia, research addressing this issue is scarce.

**Methods:**

A cross-sectional study involving 258 physicians was conducted. A scale with questions on sociodemographic aspects, level of education, satisfaction with antibiotic education received, and knowledge, attitudes, and practices was administered. The scale was designed for each item to be analyzed individually or as a total score ranging from 0 to 100 (0 being the lowest and 100 the highest).

**Results:**

31.5% of physicians rated the education received on antibiotics as fair to poor. The knowledge score was 80.1 (IQR 70.5–87.5); however, 25.2% agreed to some extent that amoxicillin is useful in treating most respiratory infections, and 15% agreed that antibiotics are effective in treating upper respiratory infections. Attitudes scored 80.2 (IQR 75.0-86.5), with 99% stating that bacterial resistance is a public health problem in Colombia, but only 56.9% considering it a problem affecting their daily practice. Practices scored 75.5 (IQR 68.8–81.2), and 71.7% affirmed that if they refuse to prescribe antibiotics to a patient who does not need them, the patient can easily obtain them from another physician. General practitioners were found to have lower scores in all three indices evaluated.

**Conclusion:**

The study reveals enduring misconceptions and concerning practices in antibiotic prescription, particularly among general practitioners. Enhancing knowledge necessitates the implementation of continuous medical education programs that focus on updated antibiotic guidelines, and resistance patterns. Fostering positive attitudes requires a culture of trust and collaboration among healthcare professionals. Practical enhancements can be realized through the establishment of evidence-based prescribing guidelines and the integration of regular feedback mechanisms. Moreover, advocating for the inclusion of antimicrobial stewardship principles in medical curricula is crucial, emphasizing the significance of responsible antibiotic use early in medical education.

**Supplementary Information:**

The online version contains supplementary material available at 10.1186/s12909-024-05354-w.

## Background

The introduction of antibiotics for the treatment of human infections has advanced the practice of medicine and has led to a significant increase in life expectancy [1]. The development of these drugs has reduced morbidity and mortality from infections, facilitating progress in areas such as oncology, surgery, orthopedics, among others [2]. However, the rise of antimicrobial resistance (AMR) threatens to impede this progress and poses significant risks to public health globally [3].

In 2019, it is estimated that there were 1.27 million deaths globally attributable to bacterial resistance. In Latin America and the Caribbean, this translates to approximately 84,300 deaths, 2,370,000 years of life lost, and 2,380,000 disability-adjusted life years [4].

Among the factors contributing to the emergence of AMR, indiscriminate antibiotic use, over-the-counter antibiotic sales without a prescription, the absence of rapid diagnostic tests at the point of care, patient and family pressure, diagnostic uncertainties, and gaps in physicians’ knowledge regarding antibiotic use and the mechanisms through which bacteria develop resistance have been identified [3, 5, 6]. Among all these factors, the inappropriate prescription of antibiotics by physicians plays a crucial role.

Inadequate antibiotic prescription has been documented in several countries. A study conducted in the U.S. revealed that antibiotics were prescribed in 10% of outpatient visits, with 25% of these prescriptions being inappropriate [5]. In Europe, antibiotic use has been identified in up to 45% of viral infections, even though this pharmacological group is ineffective in such cases [7]. Another study conducted in Lima, Peru, within highly complex hospitals, found that despite physicians’ awareness of antibiotic resistance, they believed this issue was unrelated to their professional practice [8].

Considering the aforementioned, the World Health Organization (WHO) has launched campaigns aimed at governments of various countries, urging them to take measures to preserve the effectiveness of existing antibiotics. These measures include reducing inappropriate use and decreasing the number of unnecessary prescriptions. Furthermore, the WHO has advocated for promoting medical education as a strategy to encourage appropriate antibiotic prescription and combat resistance [2]. In this context, studies on knowledge, attitudes, and practices (KAPs) are particularly crucial as they can provide essential information for researchers working in this field to recommend appropriate strategies to policymakers. Simultaneously, they enable the development of a more efficient awareness-building process and ensure that programs implemented within the stakeholder group are better tailored to their needs.

Specifically in Colombia, research on knowledge, attitudes, and practices related to the use of antibiotics and antibiotic resistance is scarce. In the case of Medellín, the second most important city in the country, investigations of this nature have been conducted among medical students, the general population, and pharmacies [9–11]. In all three groups, incorrect knowledge and inappropriate practices regarding these medications have been observed, but there have been no studies from the perspective of physicians. Physicians play a fundamental role in prescribing these drugs and educating patients to promote therapeutic adherence and proper use. Therefore, the objective of the present study is to describe the knowledge, attitudes, and practices regarding antibiotics and their resistance among physicians in Medellín and nearby municipalities (Valle de Aburrá).

## Methods

### Study design: cross-sectional

Participant Selection: The study included 258 physicians working in various hospitals in the city or nearby municipalities (Valle de Aburrá) who voluntarily agreed to participate in the research. General practitioners (those practicing non-specialized medicine), residents (practicing general medicine and undergoing specialization training), and specialists (those with high levels of training and expertise in a specific area of medicine) were included. Physicians were invited to participate through snowball sampling, which commenced in December 2022 and concluded in July 2023. Initially, the researchers shared the survey with a group of physicians, who then disseminated it to other physicians within their professional circle or connected interested individuals with the researchers via email or the researchers’ contact information. Data collection concluded after 8 consecutive days elapsed without securing new responses from doctors, despite the invitation messages and reminders sent by the researchers. In order to maximize participant recruitment and facilitate survey completion, we adapted to participants’ preferences by allowing them to complete the survey either in hard copy or online. It is important to note that it was not possible to calculate the sample size because health authorities did not have a census with the total number of physicians working in the area.

Data Collection Instrument: The questionnaire was developed in 3 stages. In the first stage, 12 representative articles were reviewed [12–23]. From these articles, the relevance of the questions was assessed and 4 articles were used as primary sources of information [12, 13, 15, 16]. In the second stage, 67 questions were included, and duplicate questions were eliminated and adapted to the Colombian health system. In the third stage, the first version of the questionnaire was reviewed by a group of experts who assessed the relevance and comprehensibility of the questions, as well as the accuracy of their translation into Spanish. This was followed by a pilot test with 20 physicians to assess utility (ease of application and processing) in which participants provided valuable suggestions on the clarity and simplicity of the questionnaire. These suggestions were carefully taken into account when formulating the final version. It should be noted that the data collected during the pilot test were not included in the final analysis of the study.

The final version of the instrument comprises 5 sections: (i) A section with questions about sociodemographic data, years of work experience, level of education (General Practitioner, Resident, Specialist, Subspecialist), and usual clinical practice setting (Emergency, Hospitalization, Outpatient Consultation, Home Care), (ii) Another section with questions about the perception of education received regarding antibiotic prescription and bacterial resistance, (iii) The knowledge index consisting of 12 items where participants expressed their opinions on a 5-level Likert scale ranging from completely disagree to completely agree, (iv) The attitudes index consisting of 12 items on which physicians also expressed their opinions on a 5-level Likert scale ranging from completely disagree to completely agree, (v) The practices index comprising 13 items on which physicians indicated the frequency of each behavior on a 5-level Likert scale ranging from never to always (Supplementary Material 1. Survey).

Information Collection: Once contact was established with the physicians, the project’s objectives were presented to them. They read and signed the informed consent, and then completed the survey. The survey was conducted anonymously and self-administered. Researchers were available to address any questions, and the time taken to respond ranged between 12 and 15 min.

Data Analysis: Information was analyzed by calculating absolute and relative frequencies for qualitative variables and summary measures for quantitative variables (position, dispersion, and central tendency). The survey was analyzed so that knowledge, attitudes, and practices items could be presented individually with their absolute and relative frequencies, as well as in an overall score for each section. For this purpose, the indices were transformed into a scale from zero (worst possible score) to 100 (best possible score) using the following formula:

$$ \begin{aligned}&\text{Total}\;\text{Score} = \left[\right(\text{obtained}\;\text{score}-\text{minimum}\;\text{possible}\;\text{score}) \\&/ (\text{maximum}\;\text{possible}\;\text{score}\\&-\text{minimum}\;\text{possible}\;\text{score}\left)\right] \times 100.\end{aligned}$$

The total score is presented with the median and interquartile range. Knowledge, attitudes, and practices were compared based on participants’ perceptions of the training received on the topic and their work experience using the Mann-Whitney U test and Kruskall-Wallis test, following verification of the non-normality assumption assessed with the Kolmogorov-Smirnov test with Lilliefors correction. Lastly, linear regressions (one for each index) were conducted to identify whether the associations found in the bivariate analysis were confounded. The variables included in the regression models were those with *p* < 0.05 in the bivariate analyses. Data were processed using SPSS version 29.0, and p-values < 0.05 were considered significant.

## Results

A total of 258 physicians were included in the study, with the majority falling between the ages of 24 and 34 (71.6%). Of the participants, 57.8% were general practitioners, and 40.3% primarily worked in emergency services. The majority had extensive work experience. Physicians were asked regarding the three most commonly prescribed antibiotics in their daily practice. The most frequently prescribed antibiotic was amoxicillin 16.3%, followed by piperacillin/tazobactam 13.6%. In the second group of antibiotics, the top choice was ampicillin 8.5%. Within the third group, the most commonly selected option was Ampicillin/sulbactam at 8.1%. Regarding education on the topic, 31.5% rated it as fair to poor, and the area they felt least prepared for by their university was interpreting antibiograms. Notably, 40.1% of physicians reported having treated patients with antibiotic-resistant infections that did not respond to therapy (Table 1).

**Table 1:** Description of the demographic, work, and perception characteristics of physicians’ education, Medellin-Colombia, 2022–2023

		n	%
Sex	Female	108	42.4
	Male	147	57.6
Age group	24–34 years	184	71.6
	34–45 years	47	18.3
	> 45 years	26	10.1
Educational stage	General physician	149	57.8
	Resident	49	19.0
	Specialist physician	60	23.3
Work experience	1 year	15	5.8
	2–5 years	73	28.4
	6–10 years	107	41.6
	> 10 years	62	24.1
Usual medical practice setting	Emergency room	104	40.3
	General Ward	83	32.2
	Ambulatory	55	21.3
	Other	16	6.2
Perception on the education received on the use of AB and AMR	Bad	8	3.1
	Average	73	28.4
	Good	155	60.3
	Excellent	21	8.2
Number of consultations per day in which antibiotics are prescribed	None	34	13.2
	1–2	114	44.2
	3–4	74	28.7
	≥ 5	36	14.0
College prepares you enough to know when to start AB therapy	Yes	234	90.7
	No	24	9.3
College prepares you enough to select the best AB for each infection	Yes	188	72.9
	No	70	27.1
College prepares you enough to understand the basic of mechanisms of AMR	Yes	158	61.2
	No	100	38.8
College prepares you enough to prescribe AB	Yes	116	45.0
	No	142	55.0
College prepares you enough to find reliable sources of information to treat infections	Yes	167	64.7
	No	91	35.3
College prepares you enough to switch from IV AB to oral AB	Yes	119	46.1
	No	139	53.9
Has treated patients with AB-resistant infections that do not respond to therapy	Never	21	8.2
	Hardly ever	35	13.6
	Sometimes	98	38.1
	Almost Always	53	20.6
	Always	50	19.5

*Note*: Some columns do not add up to 100% due to missing data. AMR: Antimicrobial Resistance. AB: Antibiotic

### Knowledge

**Fig. 1:**
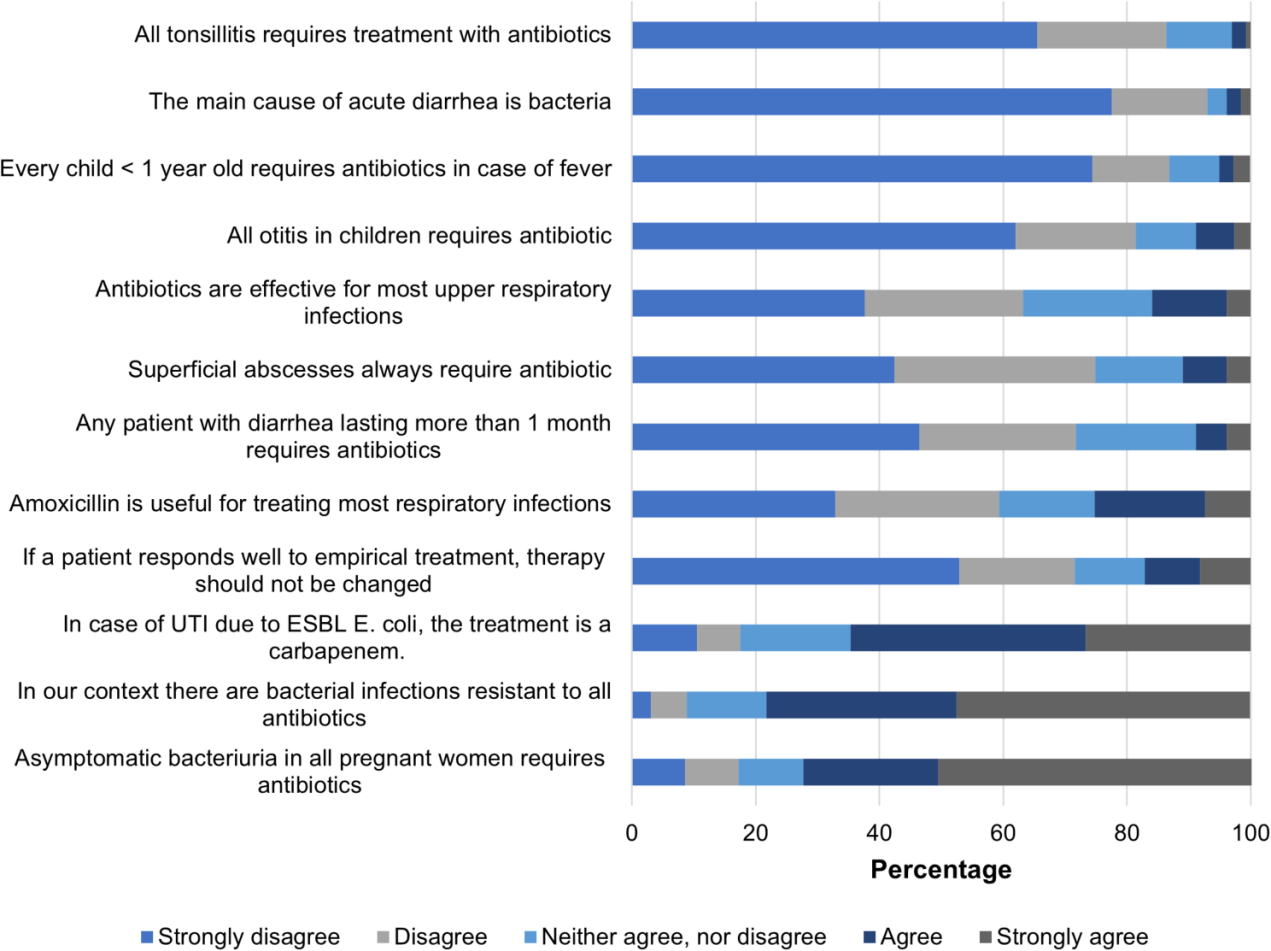
Physician responses to questions on antibiotics and bacterial resistance knowledge, Medellín-Colombia 2022–2023

In the knowledge index, it was found that 78% of physicians acknowledge the occurrence of infections caused by bacteria resistant to all available antibiotics in our context. Additionally, 25.2% show some degree of agreement that amoxicillin is useful in treating most respiratory infections, and 15% believe that antibiotics are effective in treating upper respiratory infections (Fig. 1). The overall score in this index was 80.1 (IQR 70.5–87.5), and significant differences were observed based on age group, level of education, and work experience (Table 2).

**Table 2:** Comparison of knowledge, attitudes and practices according to the demographic and work characteristics of physicians, Medellin-Colombia, 2022–2023

		Knowledge			Attitudes			Practices		
		Me	IQR		Me	IQR		Me	IQR	
Sex	Female	79.5	70.5	86.4	79.2	75.0	85.4	74.0	68.8	79.2
	Male	80.7	70.5	88.6	81.3	75.0	87.5	77.1	68.8	83.3
	P value	0.623			0.503			0.069		
Age group	24–34 years	81.8	72.7	88.6	79.2	75.0	87.5	72.9	68.8	81.3
	34–45 years	79.5	72.7	90.9	83.3	77.1	87.5	77.1	75.0	83.3
	> 45 years	63.6	59.1	79.5	79.2	68.8	83.3	76.0	68.8	79.2
	P value	0.001			0.269			0.050		
Educational stage	General physician	77.3	68.2	86.4	79.2	72.9	85.4	72.9	66.7	79.2
	Resident	86.4	75.0	88.6	81.3	77.1	89.6	77.1	70.8	83.3
	Specialist physician	79.5	70.5	86.4	81.3	75.0	91.7	79.2	72.9	85.4
	P value	0.012			0.029			0.002		
Work Experience	1 year	72.7	56.8	84.1	79.2	66.7	85.4	68.8	56.3	85.4
	2–5 years	81.8	75.0	88.6	79.2	75.0	86.5	75.0	68.8	79.2
	6–10 years	84.1	72.7	88.6	81.3	75.0	89.6	72.9	68.8	83.3
	> 10 years	77.3	63.6	84.1	81.3	75.0	85.4	77.1	72.9	81.3
	P value	0.007			0.551			0.182		
Usual practice setting	Emergency room	81.8	70.5	90.9	81.3	75.0	89.6	77.1	70.8	83.3
	General Ward	81.8	72.7	86.4	81.3	77.1	87.5	77.1	70.8	83.3
	Ambulatory	73.9	65.9	86.4	75.0	70.8	83.3	72.9	60.4	77.1
	Other	80.7	70.5	87.5	79.2	75.0	81.3	74.0	63.5	81.3
	P value	0.062			0.001			0.010		
Number of consultations per day in which antibiotics are prescribed	None	79.5	68.2	84.1	77.1	68.8	85.4	72.9	62.5	81.3
	1–2	81.8	72.7	86.4	79.2	75.0	85.4	75.0	70.8	79.2
	3–4	78.4	68.2	90.9	81.3	72.9	89.6	75.0	66.7	83.3
	≥ 5	81.8	73.9	90.9	83.3	77.1	89.6	79.2	70.8	85.4
	P value	0.249			0.139			0.185		
Has treated patients with AMR infections that do not respond to therapy	Never	72.7	65.9	86.4	77.1	66.7	81.3	68.8	62.5	75.0
	Hardly ever	79.5	68.2	84.1	79.2	70.8	83.3	70.8	64.6	75.0
	Sometimes	77.3	70.5	86.4	79.2	72.9	87.5	75.0	68.8	81.3
	Almost always	84.1	72.7	88.6	81.3	76.0	87.5	77.1	70.8	83.3
	Always	84.1	75.0	90.9	87.5	79.2	91.7	78.1	72.9	85.4
	P value	0.061			< 0.001			< 0.001		

*Note*: Me: Median. IQR: Interquartile range. AMR: Antimicrobial Resistance

### Attitudes

**Fig. 2:**
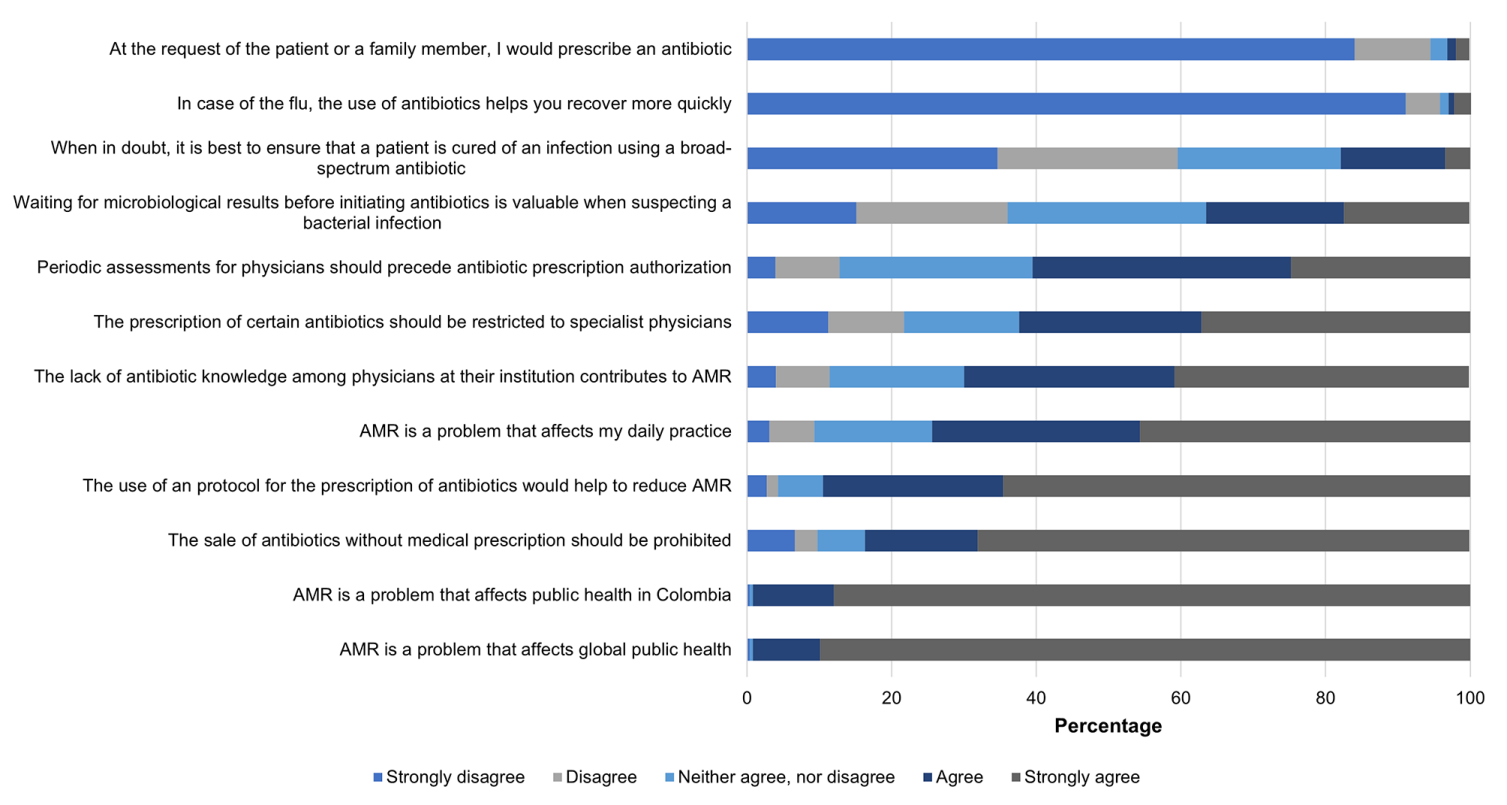
Physician responses to questions on antibiotics and bacterial resistance attitudes, Medellín-Colombia 2022–2023

In the attitudes index, it was found that 98.3% of physicians acknowledge that bacterial resistance is a problem affecting global public health, and 99% affirm that it is a problem affecting public health in Colombia. However, only 56.9% consider it a problem affecting their daily clinical practice. Notably, 60.5% believe that periodic evaluations should be implemented for physicians before allowing them to prescribe antibiotics, and 69.8% believe that the lack of knowledge among physicians in their institution contributes to the problem of bacterial resistance (Fig. 2). The score in the attitudes index was 80.2 (IQR 75.0-86.5), and significant differences were observed based on the level of education, usual clinical practice setting, whether they had treated patients with infections that did not respond to therapy (Table 2), and those who considered that the university adequately prepared them to know when to initiate antibiotic therapy (Table 3).

**Table 3:** Comparison of knowledge, attitudes and practices according to the perception of the education received about antibiotics and bacterial resistance of physicians, Medellin-Colombia, 2022–2023

		Knowledge			Attitudes			Practices		
		Me	IQR		Me	IQR		Me	IQR	
Educational perception on the use of AB and AMR	Bad	79.5	77.3	98.9	81.3	62.5	91.7	77.1	72.9	83.3
	Average	79.5	68.2	90.9	79.2	72.9	86.5	72.9	66.7	77.1
	Good	79.5	70.5	86.4	79.2	75.0	87.5	77.1	68.8	81.3
	Excellent	86.4	77.3	93.2	85.4	75.0	93.8	79.2	72.9	85.4
	P value	0.061			0.318			< 0.001		
College prepares you enough to know when to start AB therapy	Yes	79.5	70.5	88.6	81.3	75.0	87.5	75.0	68.8	81.3
	No	79.5	70.5	85.2	77.1	62.5	1.3	70.8	62.5	75.0
	P value	0.540			0.005			0.004		
College prepares you enough to select the best AB for each infection	Yes	79.5	70.5	86.4	81.3	75.0	87.5	77.1	68.8	83.3
	No	81.8	72.7	88.6	79.2	75.0	85.4	72.9	66.7	77.1
	P value	0.389			0.561			0.007		
College prepares you enough to understand the basic of mechanisms of AMR	Yes	81.8	70.5	86.4	81.3	75.0	87.5	77.1	70.8	83.3
	No	79.5	68.2	88.6	79.2	75.0	87.5	72.9	66.7	79.2
	P value	0.863			0.757			0.001		
College prepares you enough to prescribe AB	Yes	79.5	68.2	86.4	79.2	75.0	87.5	77.1	68.8	83.3
	No	81.8	72.7	88.6	81.3	75.0	87.5	75.0	68.8	79.2
	P value	0.158			0.874			0.065		
College prepares you enough to find reliable sources of information to treat infections	Yes	81.8	70.5	88.6	81.3	75.0	87.5	76.0	70.8	83.3
	No	79.5	70.5	84.1	79.2	72.9	85.4	72.9	66.7	79.2
	P value	0.131			0.115			0.021		
College prepares you enough to switch from IV AB to oral AB	Yes	81.8	72.7	88.6	81.3	75.0	87.5	77.1	70.8	83.3
	No	79.5	70.5	86.4	79.2	72.9	87.5	72.9	66.7	79.2
	P value	0.469			0.224			0.003		

*Note*: Me: Median. IQR: Interquartile range. AMR: Antimicrobial Resistance. AB: Antibiotic. IV: Intravenous

### Practices

**Fig. 3:**
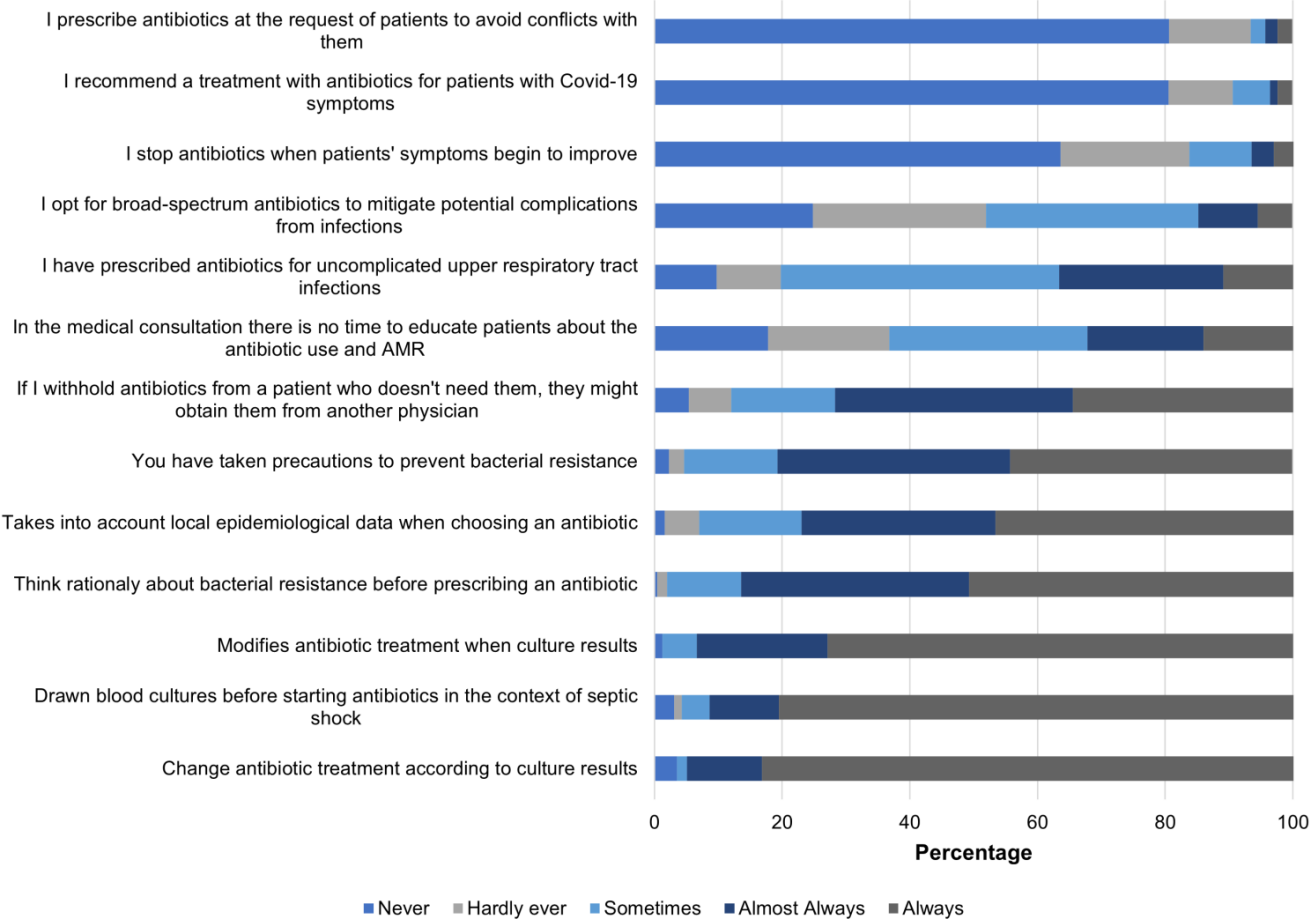
Physician responses to questions on antibiotics and bacterial resistance practices, Medellín-Colombia 2022–2023

In the practices index, it was found that 95% of physicians explain to their patients when there is no indication to use antibiotics. Additionally, 86.5% consider bacterial resistance before prescribing antibiotics, and 80.6% have taken precautions in daily clinical practice to prevent bacterial resistance. However, there is a lack of confidence in colleagues, as 71.7% believe that if they refuse to prescribe antibiotics to a patient who does not need them, the patient could easily obtain them from another doctor (Fig. 3). The score for practices was 75.5 (IQR 68.8–81.2) and showed statistically significant differences based on physicians’ level of education, usual medical practice setting, whether they had treated patients with infections that did not respond to therapy (Table 2), and their perception of the quality of education received (Table 3).

### Factors associated with KAPs

In the linear regression model, it was found that knowledge is determined by age group and level of education. Attitudes are influenced by the level of education, usual medical practice setting, treating patients with infections that do not respond to therapy, the perception of adequate preparation by the university to know when to initiate antibiotic therapy, and knowledge index. Finally, practices are influenced by treating patients with resistant infections that do not respond to therapy, university preparation for selecting antibiotics for each infection, understanding basic mechanisms of resistance, overall knowledge of the subject, and attitudes index (Table 4).

**Table 4:** Linear regression model results for physicians’ antibiotic knowledge, attitudes, and practices, Medellín-Colombia 2022–2023

		Regression Coefficients	Confidence interval 95%		P value
Knowledge	Age group	4.721	7.967	1.474	0.005
	Educational stage	2.907	0.115	4.079	0.038
Attitudes	Educational stage	1.518	0.016	3.019	0.048
	Usual medical practice setting	1.981	3.356	0.605	0.005
	Care of patients with AMR infections that do not respond to therapy	1.588	0.529	2.646	0.003
	College prepares you enough to know when to start AB therapy	10.125	14.424	5.826	< 0.001
	Knowledge	0.305	0.206	0.404	< 0.001
Practices	College prepares you enough to know when to start AB therapy	1.544	0.623	2.465	0.001
	College prepares you enough to select the best AB for each infection	3.467	5.895	1.039	0.005
	College prepares you enough to understand the basic of mechanisms of AMR	2.680	4.879	0.481	0.017
	Knowledge	0.219	0.128	0.309	< 0.001
	Attitudes	0.290	0.188	0.392	< 0.001

*Note*: AMR: Antimicrobial Resistance. AB: Antibiotic

## Discussion

This is the first study on knowledge, attitudes, and practices regarding antibiotics and their resistance conducted among physicians in Medellín and Valle de Aburrá, Colombia. The study included physicians from different medical specialties with a wide range of experience, revealing opportunities for improvement in knowledges, attitudes and practices.

First, around one-third of the participants in this study evaluated the quality of education received on antibiotics as poor or fair. Issues were also identified with the interpretation of antibiograms, understanding resistance mechanisms, and transitioning from intravenous to oral antibiotics. This finding is consistent with several previous studies where insufficient education on antibiotics has been identified [3, 24]. Inadequate education and training of healthcare professionals, including physicians, nurses, and pharmacists, are critical in this area and can lead to inappropriate antibiotic use and bacterial resistance. To address this problem, evidence-based strategies have been developed, involving low-cost multifaceted interventions, providing education to healthcare service providers, creating flowcharts for the treatment of infectious diseases, and offering on-site rapid testing [25].

In the knowledge domain, physicians harbor misconceptions concerning the efficacy of amoxicillin for treating respiratory infections, along with misguided views on the effectiveness of antibiotics in addressing upper respiratory infections. Several studies have indicated an increase in antibiotic prescriptions for the treatment of upper respiratory infections. In countries such as China, Thailand, and India, antibiotic prescriptions for upper respiratory infections exceed 70% [5, 6, 26]. However, most of these infections are viral in nature, and it has been demonstrated that antibiotics have no impact on the duration of symptoms or the severity of the illness [7]. Various factors contribute to the inappropriate prescription of antibiotics for respiratory infections, including limited time in medical consultations, poor communication between the doctor and the patient, diagnostic uncertainty, and the inability to follow up with the patient [12]. As part of the solution to this problem, Antimicrobial Stewardship Programs (ASP) have been implemented. In Colombia, the Ministry of Health, through Resolution 2471 of 2022, establishes the technical guidelines for the implementation of programs for the prevention, surveillance, and control of healthcare-associated infections and the optimization of antibiotic use [27]. As part of this strategy, there are plans to educate targeted groups and healthcare services on antimicrobial resistance, institutional-level infection treatment regimens, relevance of requests, and interpretation of laboratory tests.

Regarding attitudes, the majority of surveyed physicians acknowledge that bacterial resistance is a problem affecting global public health. However, it is noteworthy that only 56.9% consider it a problem affecting their daily clinical practice. This finding suggests that many respondents perceive antimicrobial resistance as more theoretical than practical. This result aligns with other studies where surveyed physicians consider antimicrobial resistance a significant issue but perceive it as less critical in their hospitals or usual practice settings: “Antibiotic resistance is a problem, but not in my office” [28]. The low risk perception of this problem among healthcare professionals directly impacts their practice, as underestimating the risks can lead to treatment failure, recurrent infections, healthcare-associated infections, increased mortality and morbidity rates, and higher healthcare costs [29].

Regarding practices, a high percentage of physicians express the belief that if they refuse to prescribe antibiotics to a patient who doesn’t need them, the patient can easily obtain them from another doctor. This finding reflects a lack of confidence among physicians in the practices and competencies of their colleagues. Trust is a crucial element for cooperation in the fight against bacterial resistance [30] It has been observed that individuals with trust in others, exhibit better practices because they believe that others will make decisions similar to theirs [30, 31]. Simultaneously, a lack of trust in colleagues’ practices can influence one’s own practices because “if I don’t do it, someone else will.” Promoting trust regarding the appropriate use of antibiotics is essential because trust among healthcare workers has been shown to facilitate collaboration and teamwork, thereby improving patient outcomes in the treatment of various diseases [32].In this regard, it is imperative to take measures at all levels of society to mitigate the impact and curb the spread of antibiotic resistance. To achieve a sustainable approach in this fight, education for all citizens is of vital importance. One implemented strategy is the “One Health” approach, which involves disciplines such as medicine, biology, social sciences, and education working together to achieve better results in public health [32].

Concerning the associated factors, our study identified a lower level of knowledge, attitudes, and practices (KAPs) among general practitioners. This finding is worrisome for the following reasons: (i) general practitioners constitute the largest number of medical professionals in the country, with some figures from the Ministry of Health indicating 87,000 general practitioners compared to only 28,000 specialists [33], (ii) general practitioners are the first point of contact for patients within the healthcare system. They establish the initial diagnosis, decide on the first treatment for patients, and determine if a referral to specialists is necessary, (iii) general practitioners play a crucial role in patient education, health promotion, and disease prevention. If they have deficiencies in knowledge, attitudes, and practices regarding antibiotics and bacterial resistance, these misconceptions can be transferred to patients. Additionally, it has been demonstrated that lower knowledge, attitudes, and practices related to antibiotics and resistance are associated with increased antibiotic prescriptions, difficulties in identifying resistance mechanisms, misdiagnoses, non-adherence to clinical guidelines, prolonged hospital stays, and increased healthcare costs [34]. To address this issue, continuous medical education programs, seminars, and workshops focused on updating medical knowledge are recommended. The implementation of such educational programs in some developing countries has shown to enhance the quality of diagnoses and antibiotic prescriptions [35].

This research has the following limitations: (i) the sampling was not random, as there is no census available for the number of doctors working in the area. The lack of knowledge of the finite population of physicians prevented the calculation of the ideal study sample and, consequently, the final participation rate. This is a limitation related to the statistical significance and external validity of the results, (ii) Another limitation of this study lies in the fact that some of the associations explored may suffer from beta error; however, this does not affect the comparisons in which significant associations were detected, (iii) the study is cross-sectional, meaning that associations are of a statistical nature and may not reflect causality, (iv) due to the characteristics of the questions and the study population, doctors may respond based on affective, social, and professional motivations rather than on their actual practice, leading to social desirability bias. Attempts were made to mitigate this bias by ensuring the anonymity of all participants.

## Conclusion

A high proportion of surveyed physicians perceived a poor quality of education regarding antibiotics, indicating the need to enhance training and understanding of this critical topic. General practitioners demonstrated lower levels of knowledge, attitudes, and practices compared to specialist physicians and residents. To enhance knowledge, implementing continuous medical education programs focusing on updated antibiotic guidelines, resistance patterns, and prudent prescribing practices is essential. Promoting positive attitudes requires fostering a culture of trust and collaboration among healthcare professionals, emphasizing the collective impact of responsible antibiotic use. Practical improvements can be achieved through the establishment of evidence-based prescribing guidelines, and incorporating regular feedback mechanisms. Furthermore, it is imperative to advocate for the incorporation of antimicrobial stewardship principles into medical curricula, reinforcing the importance of judicious antibiotic use from the early stages of medical education.

### Electronic supplementary material

Below is the link to the electronic supplementary material.

Supplementary Material 1

## Data Availability

The datasets used and/or analyzed during the current study are available from the corresponding author on reasonable request.
