# Supplementary material for: Understanding antibiotic knowledge, attitudes, and practices: a cross-sectional study in physicians from a Colombian region, 2023
Source: BMC Med Educ. 2024 Apr 8;24:380. doi: 10.1186/s12909-024-05354-w (PMC11000393; doi:10.1186/s12909-024-05354-w)
Supplement: Supplementary file 1 — Supplementary Material 1 [file 12909_2024_5354_MOESM1_ESM.docx]

**Survey on Knowledge, Attitudes and Practices (KAP) related to antibiotics and bacterial resistance.**

The data requested in this survey is confidential, in no case will your identity be requested and the information obtained here can only be used for investigative purposes. The results will be published through statistical aggregates. We ask you to please answer honestly to the questions that we will ask you below, remember that the response is anonymous.

| 1 Code | 2. Age |
| --- | --- |
| 3. Sex: Male ☐ Female☐ | 4. ¿How long ago did you graduate from  medical school? |
| 5. ¿What is your level of training?  General practitioner☐  Specialist: ☐ Which specialty?  Subspecialist ☐  Resident☐_________________  Other: | 6. What is the typical medical practice environment?  Emergencies☐  Hospitalization ☐  External consultation☐ Home care☐  Other: |
| 7. ¿ How would you rate your education so far regarding antibiotic use and bacterial resistance?  Excellent ☐ Good☐ Regular☐ Bad☐ I have not received education on the subject☐ | |
| 8. Approximately how many visits per day do you prescribe antibiotics?   - None ☐ Between 1 and 2 ☐ Between 3 and 4 ☐ More than 5 | |
| 9. Which antibiotics do you prescribe most frequently? Generic name, please.  _________________ _________________ _________________ _________________ | |
| 10. Do you think college prepares you enough to do the next thing after you graduate?  Know when to start antibiotic therapy Yes☐ No☐  How to select the best antibiotic for each specific infection Yes☐ No☐  Understand the basic mechanisms of antibiotic resistance Yes☐ No☐ | |
| How to interpret antibiograms Yes☐ No☐  How to find reliable sources of information to treat infections Yes☐ No☐  How to Switch from IV Antibiotics to Oral Antimicrobials Yes☐ No☐ | |
| 11. ¿ When you have doubts about choosing an antibiotic treatment, how do you resolve them?  Online sources (WEB) ☐ Smartphone applications ☐  Seminars and workshops ☐ Medical books and journals ☐  Other doctors as colleagues ☐ Representatives of pharmaceutical companies☐  Other ☐ Which other | |

Below you will find some statements, I ask you to mark from one to five if you agree/disagree with them, like this:

**1. Completely disagree, 2. Disagree, 3. Neither agree nor disagree, 4. Agree, 5.Completely agree**

|  | 1 | 2 | 3 | 4 | 5 |
| --- | --- | --- | --- | --- | --- |
| Asymptomatic bacteriuria in all pregnant women should be treated with antibiotics |  |  |  |  |  |
| In our context there are bacterial infections resistant to all antibiotics |  |  |  |  |  |
| In case of UTI due to ESBL-positive E. coli, the treatment of choice is a carbapenem. |  |  |  |  |  |
| If a patient responds well to empirical treatment, therapy should not be changed to a narrower-spectrum antibiotic |  |  |  |  |  |
| Amoxicillin is useful for treating most respiratory infections |  |  |  |  |  |
| Any patient with diarrhea lasting more than 1 month requires antibiotics |  |  |  |  |  |
| Superficial abscesses always require antibiotic management |  |  |  |  |  |
| Antibiotics are effective for most upper respiratory infections |  |  |  |  |  |
| All otitis in children requires antibiotic treatment |  |  |  |  |  |
| Every child under 1 year of age requires the use of antibiotics in case of fever |  |  |  |  |  |
| The main cause of acute diarrhea is bacteria |  |  |  |  |  |
| All tonsillitis requires treatment with antibiotics |  |  |  |  |  |

Below you will find some statements, I ask you to mark from one to five if you agree/disagree with them

as follows: **1. Completely disagree, 2. Disagree, 3. Neither agree nor disagree, 4. Agree, 5.Completely agree**

|  | 1 | 2 | 3 | 4 | 5 |
| --- | --- | --- | --- | --- | --- |
| Antimicrobial resistance is a problem that affects global public health |  |  |  |  |  |
| Antimicrobial resistance is a problem that affects public health in Colombia |  |  |  |  |  |
| The sale of antibiotics without medical recipe should be prohibited |  |  |  |  |  |
| The use of an institutional protocol for the prescription of antibiotics would help to reduce antimicrobial resistance |  |  |  |  |  |
| Antimicrobial resistance is a problem that affects my daily practice |  |  |  |  |  |
| The lack of knowledge of physicians at their institution about the use of antibiotics contributes to bacterial resistance |  |  |  |  |  |
| The prescription of certain antibiotics should be restricted to specialist physicians use only |  |  |  |  |  |
| Periodic evaluations of physicians should be implemented before allowing them to prescribe antibiotics |  |  |  |  |  |
| When I suspect a bacterial infection, it is useful to wait for the microbiological results before starting an antibiotic |  |  |  |  |  |
| When in doubt, it is best to ensure that a patient is cured of an infection using a broad-spectrum antibiotic |  |  |  |  |  |
| In case of the flu, the use of antibiotics helps you recover more quickly |  |  |  |  |  |
| At the request of the patient or a family member, I would prescribe an antibiotic |  |  |  |  |  |

Below you will find some statements about some practices, please mark according to how often you do them like

this: **1. Never, 2. Almost never, 3. Sometimes, 4. Almost always, or 5. Always**

|  | 1 | 2 | 3 | 4 | 5 |
| --- | --- | --- | --- | --- | --- |
| Change antibiotic treatment according to culture results |  |  |  |  |  |
| Drawn blood cultures before starting antibiotics in the context of septic shock |  |  |  |  |  |
| Modifies antibiotic treatment when culture results |  |  |  |  |  |
| Think rationally about bacterial resistance before prescribing an antibiotic |  |  |  |  |  |
| Takes into account local epidemiological data when choosing an antibiotic |  |  |  |  |  |
| You have taken precautions in your daily practice to prevent bacterial resistance |  |  |  |  |  |
| If I refuse to give antibiotics to a patient who doesn't need them, patient could easily get them from another physician |  |  |  |  |  |
| In the medical consultation there is no time to educate patients about the proper use of antibiotics and resistance |  |  |  |  |  |
| I have prescribed antibiotics for patients with not complicated upper respiratory tract infection |  |  |  |  |  |
| I prefer to use broad-spectrum antibiotics to avoid complications from an infection |  |  |  |  |  |
| I stop antibiotics when patients' symptoms begin to improve |  |  |  |  |  |
| I recommend a treatment with antibiotics for patients with Covid-19 symptoms |  |  |  |  |  |
| I prescribe antibiotics at the request of patients to avoid conflicts with them |  |  |  |  |  |
